# Prevalence and risk factors for impaired activities of daily living in patients with neo-vascular age-related macular degeneration who present for anti-VEGF treatment

**DOI:** 10.1038/s41433-024-02983-9

**Published:** 2024-02-19

**Authors:** Kim Van Vu, Paul Mitchell, Harshil Dharamdasani Detaram, George Burlutsky, Gerald Liew, Bamini Gopinath

**Affiliations:** 1 Centre for Vision Research, The Westmead Institute for Medical Research, The University of Sydney, Sydney, NSW Australia; 2 Macquarie University Hearing, Department of Health Sciences, Macquarie University, Sydney, NSW Australia

**Keywords:** Risk factors, Macular degeneration

## Abstract

**Background/Objectives:**

To assess the prevalence and correlates of impaired activities of daily living (ADLs) in patients with neovascular age-related macular degeneration (nAMD) who present for anti-vascular endothelial growth factor (VEGF) therapy.

**Methods:**

In a clinic-based cohort of 437 patients with nAMD who presented for anti-VEGF therapy, the Older American Resources and Services Scale (OARS) was administered to assess for impairments in basic, instrumental and total ADL. Logistic regression analyses were conducted to determine odds ratios (OR) and 95% confidence intervals (CI) for factors associated with ADL impairment.

**Results:**

The prevalence of impaired basic, instrumental and total ADL was 37.76%, 67.82% and 39.59%, respectively. In multivariate-adjusted models, moderate visual impairment [OR 5.65, 95% CI (2.31–13.83) and blindness [OR 5.43, 95% CI (2.09–14.12)] were associated with greater odds of impaired total ADL. Depressive symptoms [OR 2.08, 95% CI (1.08–4.00)], the presence of any disability [OR 3.16, 95% CI (1.64–0.07)] and never driving [OR 4.00, 95% CI (1.60–10.00)] were also positively associated with total ADL impairment. Better vision-related quality of life (QoL) was inversely associated with impaired instrumental ADL whilst higher health-related QoL scores were associated with decreased odds of total ADL impairment.

**Conclusions:**

There is a high prevalence rate of ADL impairment among nAMD patients presenting for therapy. Visual impairment, never driving, poor physical and mental health increased the odds of experiencing ADL impairment whilst better VRQoL and HRQoL reduced the odds of impairment.

## Introduction

Age-related macular degeneration (AMD) is the number one cause of visual impairment and blindness among older adults [1]. There are two types of AMD: atrophic or ‘dry’ AMD and neovascular or ‘wet’ AMD (nAMD); the latter of which may be managed using anti-vascular endothelial growth factor (anti-VEGF) therapy. In Australia, anti-VEGF drugs including aflibercept and ranibizumab are among the top ten most costly drugs under the Pharmaceutical Benefits Scheme (PBS) [2]. Yet, despite the advent of these drugs, nAMD is associated with an increased risk of impairments in activities of daily living (ADL) [3].

ADLs assess patients’ level of functional ability. Basic ADLs (BADL) such as eating, dressing and showering focus upon self-care. Instrumental ADLs (IADL) including cooking, cleaning and shopping examine one’s ability to function within society. There is a paucity of research regarding ADL impairment in nAMD patients. Taylor et al.’s [4] systematic review identified only nine studies that examined ADL impairment among AMD patients; the majority of which examined any AMD. Hochberg et al.’s [5] analysis of both wet and dry AMD subjects with bilateral or severe unilateral visual acuity loss found that those with AMD had an over 3-fold increased risk of IADL impairment. Gopinath et al.’s [6] longitudinal study reported that the presence of any AMD was a risk factor for impaired IADLs and total ADLs. In addition, late AMD increased the risk of impaired total ADL by almost 13-fold. With regards to nAMD, Creuss et al. [7] found that Canadian nAMD patients were over two times more likely to receive assistance with ADLs compared to control subjects. Similarly, Lotery et al.’s [3] UK study found that nAMD patients required more assistance with ADLs than controls. This study reported that the annual cost of nAMD patients for the NHS was seven times higher than for control subjects as these patients utilised more healthcare resources. Thus, ADL impairment has both a personal cost for patients and constitutes a significant burden upon the healthcare system.

Accordingly, research into the correlates associated with poor day-to-day functioning among nAMD patients is necessary. Such research will enable the development of targeted interventions that can improve the overall function of these patients and maximise the benefits gained from costly anti-VEGF therapies whilst reducing the overall economic cost of nAMD upon the healthcare system. Thus, our study aims to examine the prevalence of and correlates for impaired ADLs in patients with nAMD who present for anti-VEGF therapy.

## Methods

### Study population

Our study involved a clinic-based cohort of AMD patients that presented to ophthalmology clinics at either Westmead Hospital or a tertiary referral clinic (Sydney West Retina) in Sydney, Australia. Data was collected from 621 participants who were consecutively enroled between 2012 and 2015. Of these, 547 were diagnosed and treated for nAMD while the remainder were either diagnosed with early AMD or geographic atrophy (dry AMD) only. A further 110 participants were excluded as they had had incomplete ADL information and hence, 437 participants were included for cross-sectional analysis [8]. All participants provided informed consent prior to participation. This study was approved by the Western Sydney Local Health District Human Research Ethics Committee. The study adheres to the tenets of the Declaration of Helsinki.

### Assessment of AMD

For all study participants, 30° and 40° stereoscopic colour retinal photographs were taken of each eye. Cirrus spectral-domain optical coherence tomography (OCT) was utilised to assess for macular lesions. The inclusion criteria was the presence of nAMD in at least one eye, as shown by subretinal or retinal haemorrhage, pigmental epithelial or neurosensory detachment, subretinal fibrosis or old atrophic disciform of photocoagulation scars. The following clinical features of nAMD were also assessed: AMD status (early vs. late) in the better and worse eye, presence of bilateral disease, fluid type in each eye, central macular thickness and visual acuity. Patients with dry nAMD only were not included in this study. All patients within our study had presented to the eye clinic for anti-VEGF therapy. Details regarding the drug(s) used and number of injections received by each patient was also recorded.

### Assessment of activities of daily living

ADL impairment was assessed using the Older American Resources and Services Scale (OARS) [9], which examines both BADLs and IADLs. The BADLs assessed were: eating, dressing, grooming, toileting, walking and getting in and out of bed. The IADLs examined were: meal preparation, housework, shopping, telephone use, ability to take one’s medicine, travel and ability to manage one’s finances. Each of these items are scored using a 3-point scale: 0 points if an individual is unable to complete a task; 1 point if an individual may perform the task with some help; and 2 points if the individual may perform the task without any aid. Scores are then collated to produce a BADL score (between 0 and 14), IADL score (between 0 and 14) and a total ADL score (between 0 and 28). Lower scores indicate greater ADL impairment.

### Assessment of other covariates

Trained interviewers completed face-to-face interviews with each participant. During this process, information was obtained regarding participants’ sociodemographic characteristics, past medical history and lifestyle.

The presence of depressive symptoms was analysed via the Mental Health Index (MHI), which is a component of the Short-Form 36 (SF-36) scale [10], as well as the Centre for Epidemiology Studies-10-point scale (CESD-10) [11]. Cut-off scores of $\le$59 on the MHI and $\ge$10 on the CESD-10 were used to define the presence of significant depressive symptoms. The vision-related quality of life (VRQoL) of patients was assessed using the National-Eye Institute 25-Item Visual Function Questionnaire (NEI-VFQ-25) [12]. Two scales were utilised to examine health-related quality of life (HRQoL): the SF-36 and EuroQol (EQ-5D). The SF-36 assesses both the physical and psychological health of patients to produce a physical component score (PCS) and a mental component score (MCS), with higher scores indicating a better HRQoL. The EQ-5D [13, 14] includes a visual analogue scale (VAS) and a summary score.

Finally, the interviewers also assessed patients for the presence of any disability. Participants were observed for any hearing, language or speech impairment, walking difficulties, a continuous cough and dementia.

### Statistical analysis

All statistical analyses were completed using SAS software 9.4 (SAS Institute, North Carolina, USA). The baseline characteristics of participants was analysed using t-tests and chi-square tests. Age was the only continuous variable that was analysed and was of normal distribution. Logistic regression analysis was utilised to assess the association between sociodemographic, clinical, physical and mental health characteristics of participants and ADL impairment. Models were initially adjusted for age and sex. The multivariable model was then adjusted for covariates that were significant in age-sex adjusted models.

## Results

Complete information on sociodemographic characteristics, clinical features of disease, broader physical and mental health and quality of life, was obtained from 437 patients. These data were included in the final cross-sectional analysis. We found that 37.76% and 67.82% of study participants reported impairments in BADLs and IADLs respectively. The prevalence of impaired total ADLs in our sample was 39.59%. Table 1 shows that participants with impaired overall ADL were more likely to be older, not married, have poorer physical health, lead less healthy lifestyles (as indicated by their smoking status, alcohol consumption and physical activity levels) and have poor self-related health.

**Table 1:** Characteristics of study participants stratified by the presence and absence of total ADL impairment.

Characteristics	Participants (*n* = 437)		
	No impaired total ADLs (*n* = 264)	Impaired total ADLs (*n* = 173)	*p*-values
Age (years)	77.0 (8.4)	82.3 (8.4)	**<0.0001**
Female sex (%)	53.79	60.69	0.15
Married	50.76	41.62	**0.0025**
Tertiary education	21.18	14.88	0.14
$\ge$3 chronic diseases	35.11	53.76	**0.0001**
Hospital admission in past 12 months	24.43	40.46	**0.0004**
Other eye conditions	64.37	76.88	**0.0056**
Family history of AMD	20.35	17.11	0.43
Smoking status			**0.03**
- Current	9.96	5.20	
- Former	44.44	37.57	
- Never	45.59	57.23	
Alcohol consumption			**0.01**
- No alcohol	42.15	56.07	
- 0–2 standard drinks	41.76	33.53	
- >2 standard drinks	16.09	10.40	
Physical activity level (METs)			**<0.0001**
- First tertile	21.90	62.50	
- Second tertile	33.33	24.26	
- Third tertile	44.76	13.24	
Poor self-rated health	27.80	61.99	**<0.0001**

Except for age which is presented as mean (SD), all other data are presented as %.

Bold values indicate statistical significance *p* < 0.05.

*ADLs* activities of daily living, *AMD* age-related macular degeneration, *METs* metabolic equivalents.

Table 2 illustrates the associations between visual acuity, mental and physical health indices and ADL impairment. In age-sex adjusted models, the presence of visual impairment was significantly associated with poorer BADL, IADL and total ADL scores. Other clinical features of disease that were not significantly associated with ADL impairment were: AMD status in the better and worse eye, unilateral vs. bilateral disease, the presence of subretinal or intraretinal fluid or pigment epithelial detachment in the better and worse eye, central macular thickness and the number of anti-VEGF injections received.

**Table 2:** Association between visual acuity, physical and mental health indices and the presence of ADL impairment in nAMD patients.

	ADL scores		
	Impaired BADLs Age/sex adjusted OR (95% CI)	Impaired IADLs Age/sex adjusted OR (95% CI)	Impaired total ADLs Age/sex adjusted OR (95% CI)
*Visual acuity*			
- Normal VA (>20/40)	1.0 (Ref.)	1.0 (Ref.)	1.0 (Ref.)
- Mild VI ($\le$20/40 to >20/80)	1.29 (0.77–2.18)	**1.77 (1.05–2.98)**	**1.75 (1.03–3.00)**
- Moderate VI ($\le$20/80 to >20/200)	**3.34 (1.89–5.90)**	**5.28 (2.52–11.07)**	**6.22 (3.42–11.31)**
- Legally blind ($\le$20/200)	1.81 (0.95–3.48)	**6.50 (2.42–17.45)**	**6.11 (3.03–12.32)**
*Mental health*			
Presence of depressive symptoms (CESD-10)	**3.77 (2.47–5.77)**	**4.60 (2.76–7.66)**	**5.51 (3.52–8.60)**
Presence of depressive symptoms (MHI)	**3.02 (1.94–4.70)**	**5.63 (3.10–10.25)**	**5.77 (3.57–9.32)**
*Physical health*			
$\ge$3 chronic diseases	**1.51 (1.01–2.27)**	1.28 (0.83–1.97)	**1.99 (1.32–3.00)**
Hospital admission past 12 months	**2.35 (1.52–3.62)**	**2.03 (1.23–3.35)**	**1.88 (1.22–2.89)**
*Disability status*			
Any disability	**2.98 (1.92–4.60)**	**4.26 (2.49–7.31)**	**4.48 (2.87–6.99)**
Hearing	**2.19 (1.17–4.09)**	**3.44 (1.30–9.13)**	**2.73 (1.42–5.25)**
Speech/language	**1.98 (1.11–3.51)**	**3.59 (1.61–7.99)**	**3.41 (1.85–6.27)**
Walking	**4.73 (2.86–7.84)**	**5.15 (2.46–10.82)**	**4.38 (2.64–7.27)**
Dyspnoea	1.68 (0.23–12.43)	>999.999 (<0.001–>999.999)	1.50 (0.21–10.93)
Dementia	**3.97 (1.23–12.84)**	>999.999 (<0.001–>999.999)	**8.26 (1.82–37.46)**
*Driving status*			
Current	1.0 (Ref.)	1.0 (Ref.)	1.0 (Ref.)
Previous	**2.63 (1.50–4.61)**	**3.97 (2.31–6.81)**	**7.13 (3.79–13.42)**
Never	**3.88 (2.15–6.99)**	**8.99 (4.56–17.76)**	**10.64 (5.38–21.02)**

Bold values indicate statistical significance *p* < 0.05.

*BADLs* basic activities of daily living, *IADLs* instrumental activities of daily living, *ADLs* activities of daily living, *OR* odds ratio, *CI* confidence interval, *CESD-10* Centre of Epidemiology Studies Depression-10 scale, *MHI* Mental Health Index.

As shown in Table 2, in age-sex adjusted models, the presence of depressive symptoms, a hospital admission within the past 12 months, the presence of any disability and giving up or never driving were significantly associated with impaired BADLs, IADLs and total ADLs. In addition, the presence of $\ge$3 chronic diseases was associated with impaired BADLs and total ADLs.

Table 3 shows that in age-sex matched models, lower VRQoL scores were associated with impairments in BADLs, IADLs and total ADLs. Similarly, lower physical and mental components scores plus lower scores on the EQ-5D-5L visual analogue scale and summary scores were associated with poorer functional ability.

**Table 3:** Association between quality of life measures and the presence of ADL impairment in nAMD patients.

	Baseline		
	*Impaired BADLs* Age/sex adjusted OR (95% CI)	*Impaired IADLs* Age/sex adjusted OR (95% CI)	*Impaired total ADLs* Age/sex adjusted OR (95% CI)
*Vision-related QoL*			
*NEI-VFQ-25*			
- First tertile	**4.35 (2.55–7.42)**	**23.97 (10.75–53.43)**	**17.62 (9.22–33.64)**
- Second tertile	1.56 (0.91–2.67)	**4.06 (2.44–6.76)**	**4.04 (2.15–7.59)**
- Third tertile^a^	1.0 (Ref.)	1.0 (Ref.)	1.0 (Ref.)
*Health-related QoL*			
*SF-36 PCS*			
- First tertile	**7.42 (4.06–13.56)**	**11.41 (5.80–22.43)**	**8.05 (4.32–14.99)**
- Second tertile	**2.91 (1.59–5.33)**	**4.87 (2.78–8.54)**	**4.25 (2.29–7.87)**
- Third tertile^a^	1.0 (Ref.)	1.0 (Ref.)	1.0 (Ref.)
*SF-36 MCS*			
- First tertile	**4.12 (2.40–7.09)**	**5.71 (3.00–10.86)**	**5.46 (3.12–9.55)**
- Second tertile	1.29 (0.74–2.25)	1.48 (0.87–2.51)	1.02 (0.58–1.80)
- Third tertile^a^	1.0 (Ref.)	1.0 (Ref.)	1.0 (Ref.)
*EQ-5D-5L VAS*			
- First tertile	**17.49 (9.23–33.12)**	**26.30 (11.66–59.30)**	**22.48 (11.62–43.49)**
- Second tertile	**2.62 (1.40–4.91)**	**4.08 (2.42–6.87)**	**2.91 (1.55–5.48)**
- Third tertile^a^	1.0 (Ref.)	1.0 (Ref.)	1.0 (Ref.)
*EQ-5D-5L summary score*			
- First tertile	**5.04 (2.95–8.61)**	**17.17 (7.75–38.03)**	**8.27 (4.70–14.55)**
- Second tertile	**2.11 (1.23–3.63)**	**2.20 (1.31–3.70)**	**1.76 (1.02–3.06**
- Third tertile^a^	1.0 (Ref.)	1.0 (Ref.)	1.0 (Ref.)

Bold values indicate statistical significance *p* < 0.05.

*BADLs* basic activities of daily living, *IADLs* instrumental activities of daily living, *ADLs* activities of daily living, *OR* odds ratio, *CI* confidence interval, *QoL* quality of life, *NEI-VFQ-25* National Eye Institute Visual Function Questionnaire-25, *SF-36 PCS* Short-Form 36 Physical Component Score, *SF-36 MCS* Short-Form 36 Mental Component Score, *EQ-5D-5L EuroQoL* VAS Visual Analogue Scale.

^a^The third tertile of scores has been used as the reference point as higher scores indicate better QoL.

The multivariable adjusted model is presented in Table 4. Moderate VI and blindness (over 5-fold greater odds), the presence of depressive symptoms, the presence of any disability and never driving were significantly associated with greater odds of total ADL impairment whilst, higher PCS and VAS scores were protective against ADL disability. In addition, having a hospital admission within the past 12 months was associated with an over two-fold increased risk of BADL impairment whilst better VRQoL was associated with a lower risk of IADL impairment.

**Table 4:** Multivariable-adjusted associations for impaired activities of daily living in individuals with nAMD.

	Prevalence of BADL impairment	Prevalence of IADL impairment	Prevalence of total ADL impairment
	OR (95% CI)	OR (95% CI)	OR (95% CI)
*Age (1-year increase)*	1.03 (0.99–1.06)	**1.04 (1.01–1.08)**	1.02 (0.98–1.05)
*Gender*	0.57 (0.32–1.04)	0.91 (0.49–1.67)	1.50 (0.78–2.89)
*Visual acuity*		Not significant	
*- Normal*	1.0 (Ref.)		1.0 (Ref.)
*- Mild VI*	0.95 (0.47–1.94)		1.31 (0.61–2.81)
*- Moderate VI*	**3.47 (1.55–7.79)**		**5.65 (2.31–13.83)**
*- Blind*	0.82 (0.34–1.99)		**5.43 (2.09–14.12)**
*Presence of depressive symptoms (CESD-10)*	1.75 (0.96–3.19)	Not significant	**2.08 (1.08–4.00)**
$\ge$3 chronic diseases	Not significant*	Not significant	Not significant
Hospital admission past 12 months	**2.08 (1.16–3.76)**	Not significant	Not significant
*Presence of any disability*	Not significant*	**2.25 (1.03–4.90)**	**3.16 (1.64–6.07)**
*Driving status*			
*- Current*	1.0 (Ref.)	1.0 (Ref.)	1.0 (Ref.)
*- Former*	1.28 (0.58–2.81)	0.97 (0.44–2.15)	2.29 (0.96–5.45)
*- Never*	**2.51 (1.12–5.62)**	**3.97 (1.70–9.26)**	**4.00 (1.60–10.00)**
*Vision-related QoL*	Not significant*		Not significant
*- First tertile*		1.0 (Ref.)	
*- Second tertile*		**0.18 (0.06–0.53)**	
*- Third tertile*		**0.07 (0.02–0.21)**	
*SF-36 PCS*			
*- First tertile*	1.0 (Ref.)	1.0 (Ref.)	1.0 (Ref.)
*- Second tertile*	0.55 (0.30–1.03)	0.46 (0.20–1.06)	0.72 (0.36–1.42)
*- Third tertile*	**0.32 (0.14–0.75)**	0.13 (0.06–0.29)	**0.25 (0.10–0.63)**
*EQ-5D-5L VAS*		Not significant	
*- First tertile*	1.0 (Ref.)		1.0 (Ref.)
*- Second tertile*	**0.25 (0.13–0.48)**		**0.18 (0.09–0.38)**
*- Third tertile*	**0.23 (0.09–0.58)**		**0.23 (0.09–0.60)**

Bold values indicate statistical significance *p* < 0.05.

*BADLs* basic activities of daily living, *IADLs* instrumental activities of daily living, *ADLs* activities of daily living, *OR* odds ratio, *CI* confidence interval, *VI* visual impairment, *CESD-10* Centre of Epidemiology Studies Depression-10 scale, *QoL* quality of life, *SF-36 PCS* Short-Form 36 Physical Component Score, *SF-36 MCS* Short-Form 36 Mental Component Score, *EQ-5D-5L* EuroQoL, *VAS* Visual Analogue Scale.

## Discussion

Our study found a high prevalence of ADL impairment among nAMD patients; a finding that correlates with previous studies [5, 6]. However, whilst previous studies had significantly smaller samples and often only analysed IADL impairment, we have used a large, clinic-based cohort of patients to show that BADL, IADL and total ADL impairment are all highly prevalent among nAMD patients. Hence, our study is an important contribution to the existing evidence-base in this area. These findings suggest that nAMD adversely impacts the ability of a large number of patients to undertake everyday tasks. This justifies the need for research that identifies the risk factors for such impairments and the development of targeted interventions that will support nAMD patients to function within society.

We found an association between VA and ADL impairment. This correlates with Lotery et al.’s [3] research, which reported that 35% of nAMD with “severe VA” and 66.7% with “near-blindness” required ADL-related assistance. However, this study analysed patients with bilateral nAMD only, whilst it is unclear how ADL impairment was measured. In contrast, our research utilises a larger sample and a validated ADL scale to show an independent association between poor VA and ADL impairment. This is unsurprising, as poor vision is likely to impact one’s ability to execute daily tasks. We found similar associations between moderate VI and blindness with ADL impairment, these observed associations between VA and ADL impairment highlights the importance of preserving sight in nAMD patients in order to mitigate the risk of possible ADL disability.

Our study presents a novel association between the presence of depressive symptoms as per the CESD-10 scale and total ADL impairment in nAMD patients. This association between depressive symptoms and ADL impairment has been previously described in the broader geriatrics literature [15]. Mehta et al. [16] suggest that depressive symptoms may adversely affect the physical skills required to maintain functional independence whilst also rendering these individuals less resilient to acute stressors such as poor health. Together, these factors may cause individuals to become more susceptible to deteriorations in their functional status. Interestingly, we previously reported an association between depressive symptoms and ADL impairment [17]. It is also noteworthy that poor MCS was not associated with ADL impairment among nAMD patients. Thus, the CESD-10 and the MCS, which contains the MHI scale, rendered differing results; a finding that is in keeping with previous studies [17]. As Gopinath et al. [18] explains, it is possible that these scales measure different aspects of mental health, thereby, accounting for the variations in results.

Our study highlights the effect of patients’ broader physical health upon their level of functioning. Hospitalisation within the past 12 months was associated with BADL impairment whilst the presence of any disability was associated with impairments in IADLs and total ADLs. This is consistent with the geriatrics literature, which has identified hospitalisation to be a risk factor for ADL impairment as well as subsequent nursing home placement and death [19]. Gill et al. [20] explain that illnesses that are sufficiently serious to result in hospitalisation may cause and/or worsen functional impairments whilst also acting as a barrier to recovery. Similarly, disabilities such as cognitive [21] and hearing impairments [22] are independently associated with ADL impairment in older persons. Such impairments may also limit the ability of individuals to develop adaptive behaviours that offset the effects of nAMD thereby explaining their poorer functional status. It is noteworthy that doctors already routinely ask patients regarding their medical history whilst the disabilities analysed in our study such as walking impairments are easily identifiable when examining patients. Thus, our findings contribute to clinical medicine by enabling ophthalmologists to utilise information that is already routinely obtained to identify patients that are at increased risk of ADL impairment and may require additional support services.

We found an association between individuals that have never been drivers and impairments in BADLs, IADLs and total ADLs. The geriatrics literature has identified never driving to be a risk factor for needing to enter a long-term care home that offers support with ADLs such as services for meals and transportation [23]. Where cars offer older adults greater mobility, not driving may limit IADLs such as grocery shopping and reduce individuals’ abilities to engage in activities outside of the home [24]. Such inactivity then results in further physical and mental decline [25] plus higher levels of disability [26] thereby explaining the association between not driving and poor ADL status. Interestingly, we found no association between driving cessation and ADL impairment. This contrasts the geriatrics literature, which has found driving cessation to be a risk factor for both requiring long-term care and death [23, 25]. Such findings are understandable given that the risk factors for driving cessation, such as physical or cognitive decline, are also risk factors for poor functioning [25]. However, these studies did not analyse nAMD patients specifically. Thus, future research in nAMD may wish to revisit this relationship between driving cessation and ADL impairment, perhaps through the use of a longitudinal analysis, to determine whether driving cessation can be used as a surrogate marker for assessing functional decline in nAMD patients.

Our study identified that better visual function was protective against IADL impairment whilst the relationship with BADL impairment was not significant. This is unsurprising as the NEI-VFQ-25 questionnaire contains more questions pertaining to IADLs than BADLs. Whilst BADLs such as dressing and walking are addressed, there are extensive questions pertaining to IADLs. For example, being able to do “work or hobbies that require you to see well up close, such as cooking, sewing, fixing things around the house” is relevant to housework, “reading ordinary print” is necessary for both taking medicine and managing finances whilst the multiple questions regarding driving are relevant to travel. Thus, where the NEI-VFQ-25 assesses a number of higher-level skills, it is plausible that higher scores on this questionnaire are associated with lower levels of IADL impairment.

Finally, we found that higher SF-36 PCS and EQ-5D-5L VAS scores were inversely associated with ADL impairment. We have previously reported within a longitudinal study that ADL impairment is associated with poorer HRQoL in nAMD patients receiving treatment [27]. This is plausible as higher levels of function likely enables individuals to derive greater life satisfaction thereby accounting for their better HRQoL, and potentially translating to larger gains in quality adjusted life years (QALYs) - a measure used in health economics to quantify the impact of a health condition on both the quantity and QoL. However, our dataset did not offer sufficient numbers at follow-up to conduct a longitudinal analysis of the inverse relationship. Thus, future studies involving a longitudinal study design are necessary to confirm whether higher HRQoL scores are also a protective factor against ADL impairment.

The strength of the current study arises from our analysis of a large, clinic-based cohort of nAMD patients presenting for anti-VEGF therapy. We collected extensive data regarding participants’ clinical features of disease, physical and mental health and QoL. Consequently, we were able to identify and adjust for a number of confounding factors to reduce the risk of bias. Unlike previous studies, our research used validated questionnaires to assess both ADL impairment and risk factors such as the presence of depressive symptoms, VRQoL and HRQoL. A key weakness of our study is its cross-sectional study design. Hence, we are unable to determine the direction of causality of the trends identified. Further, the use of multiple questionnaires may have resulted in survey fatigue, which in turn reduces the reliability of our results. Finally, given the older demographic of our patient population, our study could be improved through the use of a validated questionnaire such as the Mini-Mental State Examination (MMSE) when assessing for the presence of dementia. This is important as cognitive impairment may have affected participants’ survey responses and thus the trends identified.

Overall, we found high rates of impaired BADLs, IADLs and total ADLs among nAMD patients presenting for anti-VEGF therapy. Factors independently associated with ADL impairment include visual acuity, driving status, the physical and mental health of patients plus poorer VRQoL and HRQoL. Where the existing research has focused upon identifying an association between AMD and ADL impairment [5], our research is novel as it identifies key correlates for ADL impairment among nAMD patients presenting for anti-VEGF therapy. For instance, the EQ-5D-5L-VAS was strongly and independently associated with overall ADL disability in nAMD patients, and it is relatively a simple and quick tool to administer in the clinic setting. Therefore, these findings contribute to existing clinical practice by better enabling ophthalmologists to identify patients at risk of ADL impairment. In addition, our study may inform the development of interventions that aim to preserve function and/or rehabilitate nAMD patients presenting for anti-VEGF therapy. This in turn, will reduce the personal and economic burden of nAMD.

## Summary

### What was known before

- Prior research has identified an association between age-related macular degeneration (AMD) and activities of daily living (ADL) impairment.
- The majority of studies have shown an association with presence of any AMD lesions and greater odds of ADL impairment.

### What this study adds

- Our research is novel as it identifies key correlates for ADL impairment among neovascular AMD (nAMD) patients presenting for anti-VEGF therapy.
- Correlates include visual acuity, driving status, the physical and mental health of patients plus poorer quality of life.
- These findings contribute to existing clinical practice by better enabling ophthalmologists to identify patients at risk of ADL impairment.

## Data Availability

Deidentified participant data will be made available on reasonable request made to the corresponding author.
